# Coping with COVID-19: a prospective cohort study on young Australians' anxiety and depression symptoms from 2020–2021

**DOI:** 10.1186/s13690-024-01397-z

**Published:** 2024-09-26

**Authors:** Ana Orozco, Alexander Thomas, Michelle Raggatt, Nick Scott, Sarah Eddy, Caitlin Douglass, Cassandra J. C. Wright, Tim Spelman, Megan S. C. Lim

**Affiliations:** 1 Disease Elimination Program, Burnet Institute, Melbourne, Australia; 2 Monash School of Public Health and Preventive Medicine, Monash University, Melbourne, Australia; 3 Melbourne School of Population and Global Health, University of Melbourne, Melbourne, Australia; 4 Menzies School of Health Research, Darwin, Australia; 5 Centre for Alcohol Policy Research, Melbourne, Australia

**Keywords:** Coronavirus, Mental health, Young people, Depression, Anxiety, Lockdown, Pandemic

## Abstract

**Background:**

Studies have shown that the coronavirus (COVID-19) pandemic negatively impacted the mental health of young Australians. However, there is limited longitudinal research exploring how individual factors and COVID-19 related public-health restrictions influenced mental health in young people over the acute phase of the COVID-19 pandemic. This study aimed to identify risk and protective factors associated with changes in individual symptoms of anxiety and depression among young Australians during the COVID-19 pandemic.

**Methods:**

This prospective cohort study collected data on anxiety and depression symptoms of young Australians aged 15–29 years old using the Depression, Anxiety and Stress Scale short form (DASS-21). We delivered four online questionnaires from April 2020 to August 2021 at intervals of 3, 6, and 12 months after the initial survey. We implemented linear mixed-effects regression models to determine the association among demographic, socioeconomic, lifestyle and COVID–19 public health restrictions related factors and the severity of anxiety and depression symptoms over time.

**Results:**

Analyses included 1936 young Australians eligible at baseline. There was a slight increase in DASS-21 anxiety mean scores from timepoint 3 to timepoint 4. DASS-21 depression scores showed slight fluctuations across timepoints with the highest mean score observed in timepoint 2. Factors associated with increases in anxiety and depression severity symptoms included LGBTQIA + identity, financial insecurity both before and during the pandemic, higher levels of loneliness, withdrawal or deferral of studies, spending more time on social media, and difficulties to sleep. Risk factors for only depression symptoms include unemployment during COVID-19 pandemic and being in lockdown. Living with someone was a protective factor for both anxiety and depression symptoms, pre-COVID-19 unemployment for depression symptoms, and older age and unemployment during the pandemic for anxiety symptoms.

**Conclusion:**

These findings indicate that during the first year of the pandemic in Australia, there were significant changes in young people’s mental health which were associated with multiple demographic, socioeconomic, lifestyle, and lockdown factors. Hence, in future public health crises, we suggest more inclusive guidelines that involve young people in their development and implementation ensuring that their unique perspectives and needs are adequately considered.

**Supplementary Information:**

The online version contains supplementary material available at 10.1186/s13690-024-01397-z.

**Table:** 

Text box 1. Contributions to the literature
• Limited longitudinal research has explored individual factors and COVID-19 related public-health restrictions influence on young Australians’ mental health over COVID-19.
• There was a significant variability in anxiety and depression severity symptoms between young participants from April 2020 to August 2021 amidst the COVID-19 pandemic. Multiple risk and protective factors were identified. Being in lockdown was a risk factor for an increase in depression symptoms. Unemployment during the pandemic was a protective factor for increased anxiety symptoms.
• Future pandemic preparedness should plan for more inclusive guidelines, involving young people in their development and implementation to ensure feasible, effective and equitable lockdowns for young people.

## Introduction

Globally, the experience of the coronavirus (COVID-19) pandemic and related public health restrictions has been associated with adverse mental health outcomes [1–7]. In Australia, mental health was already a leading concern for young people prior to the pandemic [8]. National data show that young Australians were disproportionately impacted by COVID-19 stressors including disruptions to employment and education [9, 10]. These stressors were further compounded by reduced social support [10].

Multiple systematic reviews of global cross-sectional and longitudinal studies [7, 11–21] have reported either high prevalence or significant increases in anxiety and depression symptoms in adolescents and young adults during the pandemic [22–26], with younger people more affected than older generations [27]. Some studies have found that this relationship varies based on demographic, behavioural, and social factors. Cross-sectional studies have found that young people, especially females [13, 20, 22, 28, 29], nonbinary people [5, 21], and LGBTQIA + people [30, 31], experienced higher anxiety, and depression symptoms. Additional risk factors contributing to these symptoms included living alone or with parents [13, 16] [30], unemployment [32], financial insecurity [13], disruptions in education [19], lower education [22, 30, 33], extensive screen or internet use [7, 17], levels of loneliness [16], sleep disturbances [19, 29], and COVID-19-related factors like perceived risk [11], diagnosis or suspected infection [17], and mandatory quarantine [7, 11]. Equally, protective factors identified among youth and adult samples included higher level of education, financial security, being in a relationship [34], physical activity [35], and routine [24].

Some longitudinal and repeated cross-sectional research has also been conducted in adolescent and adult populations to identify correlates of changes in anxiety and depression. Female gender [25], disruptions in education [36] increased social media use or consumption of COVID-19 media [24] and stricter lockdown mandates [37] were identified as increasing risk, while feeling socially connected was identified as protective. Despite substantial research on longitudinal factors associated with depression and anxiety during the pandemic, inconsistencies have been reported across studies [26]. Additionally, few studies to date have specifically focused on both adolescents and young adults. This age group represents a key population of interest, considering the substantial disruptions to psychosocial development during the pandemic [38].

Australia’s experience of the COVID-19 pandemic contrasted from many other countries, with strong public health restrictions translating to low case numbers in 2020 [39]. Moreover, the severity of restrictions and number of COVID-19 cases differed substantially between Australian states in 2020 and 2021. For instance, Victoria (VIC) and New South Wales (NSW) experienced multiple prolonged lockdowns in 2020 and 2021 compared to the other states that did not experience major disruptions to daily life [40]. While Meyer et al. (2023) [41] reported no significant difference in mental health (as measured by the DASS-21) between young people aged 16–24 living in Victoria and Queensland (QLD) during mid-2021, other global studies have emphasised greater negative mental health impacts of stricter lockdowns [37, 42]. Considering these mixed findings, further comparison of mental health impacts between jurisdictions throughout the pandemic is warranted.

Research in Australia identified notably elevated levels of anxiety and depression symptoms during the COVID-19 pandemic. Comparison with pre-pandemic Australian national mental health data is limited, as the last version available before the pandemic was published in 2007, and the most recent data is from during the pandemic (2020–2022) [43]. However, compared to other international community-based samples of adults studied before 2020 [44, 45], anxiety and depression levels in Australia were significantly high, particularly on younger people [5, 44, 46]. An online survey in June 2020 with a sample of 760 Australian adolescents (aged 12–18 years) showed that 48% presented psychological distress scores above the threshold indicative of mental illness [46]. A longitudinal cohort study with young Australian adults (mean age 22 years) reported a significant increase in young people’s anxiety and depression mean scores from August 2019 (pre-pandemic) to May–June 2020 (during the pandemic) [15]. Mean scores provide an idea of the average impact on the population’s anxiety and depression symptoms. However, by using measurements that acknowledge individual effects, additional and more precise insights can be learned about the impact within individuals. This will allow for further exploration of individual-level factors associated with changes in anxiety and depression symptoms during the pandemic, as supported by Witteveen et al. (2023) [26].

### Aims

This prospective cohort study aimed to investigate risk and protective factors associated with changes in the severity of anxiety and depression symptoms in young people in Australia during the COVID-19 pandemic (April 2020 to August 2021).

## Methods

### Study design and participants

**Fig. 1:**
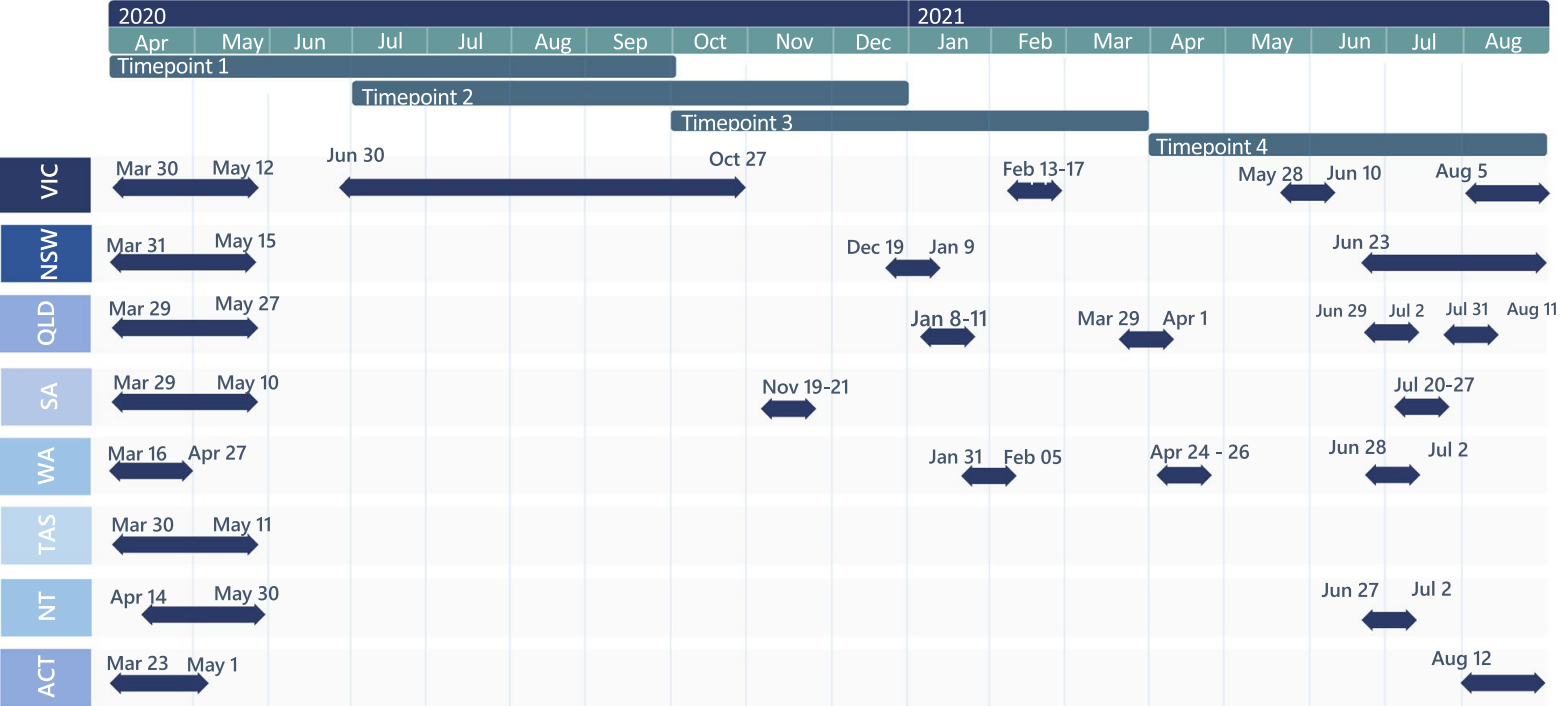
Australian lockdown dates by state and recruitment period for young people at data collection timepoints 1–4. Note: Australian states and territories: Victoria (VIC), New South Wales (NSW), Queensland (QLD), South Australia (SA), West Australia (WA), Tasmania (TAS), Northern Territory (NT) and Australian Capital Territory (ACT). Timepoint 1 refers to baseline survey when participants were recruited, and timepoint 2,3,4 to follow up surveys

This quantitative prospective cohort study recruited 2006 Australian residents aged 15–29 years to complete a baseline and three follow-up surveys over a 12-month period (see Fig. 1). This study used quota sampling to ensure minimum representation of gender, age groups and state and territories proportional to population size [47]. Quotas for states and territories were determined by the target population's demographics. Age and gender quotas allocated around 300 positions for each age-gender group along with an additional 200 slots for participants in any other category in case a group exceeds its quota, or participants identified as nonbinary gender or other genders.

### Recruitment and follow up

Participants were recruited via a market research panel Pure Profile [48] and paid social media advertising including Facebook, Instagram, Reddit, and Twitter. People recruited via social media entered a draw to receive one of five $50 vouchers per completed survey starting from the first survey. Pure Profile participants were reimbursed $3.80 for their first survey. For subsequent surveys, Pure Profile participants were entered in the draw to win $50. Participants aged 15–17 years were required to complete a mature minor comprehension form to confirm capacity to provide informed consent. Refer to Fig. 1 for recruitment periods alongside Australian lockdown dates per state.

### Data collection and questionnaire

The online survey included demographic (gender, age, sexual identity, Aboriginal or Torres Strait Islander status, relationship status), socioeconomic (living in a bushfire affected postcode, residential status, living situation, education level, employment and student status, financial security), lifestyle (social media usage, sleeping problems), and mental health (anxiety, depression, loneliness) questions. The survey was hosted on REDCap [49].

### Outcome variables

The primary outcomes of this study were the severity of anxiety symptoms and depression symptoms over the observed period. Severity of symptoms was measured using the validated and reliable short form of the Depression, Anxiety, and Stress Scale (DASS-21). [50]. The DASS-21 scores measure the continuum of severity of the core symptoms of depression, anxiety and stress; it is not intended as an anxiety and depression diagnostic tool [51]. The DASS-21 has three subscales with 21 items in total. Each item is rated on a 4-point scale from 0 "Did not apply to me at all" to 3 "Applied to me very much, or most of the time". Item scores were summed to produce subtotal scores for each subscale (7 items per subscale) and multiplied by 2, ranging from 0 to 42 points. Higher scores indicate more severe mental health symptoms.

This study focuses on the depression and anxiety subscales of the DASS-21 (i.e., 14 out of 21 items), due to their strong validity and reliability in adults [50]. The DASS-21 stress scale was excluded due to concerns about its inconsistent performance and validity, particularly in adolescent populations and in longitudinal studies [51–53]. In accordance with DASS guidelines [51] and Laranjeira et al. (2023) [54], participants at each time point with up to one missing item per subscale (*n* = 70) had the missing item replaced with the calculated mean score from the 6 remaining subscale items. Participants with more than one missing item per subscale were excluded from analysis (*n* = 33, 1.7%) (37).

### Independent variables – potential risk and protective factors

Relevant variables were classified as either static or time-varying variables. Static variables included variables at baseline such as gender (female, male, non-binary), age group (15–19, 20–24, and 25–29 years), bushfire affected postcode (no, yes), LGBTQIA + (no, yes, missing), residential status in Australia (citizen, permanent resident, other temporary visa), Aboriginal or Torres Strait Islander (no, yes), highest completed or enrolled level of education (high school, tertiary education, missing or I don’t know), work status before the pandemic (full-time, part-time, casual, unemployed, other), financial security before the pandemic (financially secure, financially insecure), recruitment approach (research market panel, social media), and loneliness (mild loneliness or lower, moderate loneliness or higher, missing data). Time-varying variables were updated at each data collection point, including baseline and follow-up surveys, such as hours spent on social media per day, living situation (alone, parents, partner, friends/roommates, other), in a relationship (no, yes, prefer not to say), student status (not a current student, going to school/university/class in person, studying, by distance/online, deferred/withdrawn/dropped studies), current work status (full-time, part-time, casual, unemployed, other), financial security when taking the survey (financially secure, financially insecure), in lockdown (no, yes), days per week having trouble to sleep (zero to two days per week, over two days per week, missing).

A “lockdown” variable was generated to reflect stay-at-home orders for each Australian State between 2020 and 2021, see specific dates in Fig. 1. Participants were coded as being in lockdown if their corresponding state was under stay-at-home orders when the participant completed each survey. Dates were collected from the state premier media announcements [40], confirmed against online news and compared against the Oxford Covid-19 Government Response Tracker (OxCGRT) database [55]. Loneliness was measured with the UCLA loneliness scale short form (ULS -6) [56]. This scale contains six questions with a score range from 6 to 24 points, where higher scores are indicative of higher levels of loneliness. We created a "bushfire" variable by identifying Australian postcodes impacted during the severe and uncontrolled bushfire season that Australia endured from September 2019 to March 2020, see specific dates in Additional file 1 [57].

### Statistical analysis

To describe and summarise the data collected we used frequencies, percentages, and mean values. We used boxplots to compare DASS- 21 anxiety and depression scores over the observation period for those not in lockdown and in lockdown, showing distribution differences.

To investigate risk and protective factors associated with changes in mental health symptoms, and account for the repeated measures within participants, we analysed DASS-21 anxiety and depression scores using mixed-effects models (Estimation method: Maximum likelihood, Fixed effects: predictors in Table 1, Random effect: Participant ID).We used two linear mixed-effects regression models fitted separately for 1) anxiety and 2) depression scores. Assumptions for both mixed-effects models were met including linearity, homoscedasticity, and normality of residuals. Random effects were checked for normality and independence, with no violations detected. Covariates were selected for inclusion in each model by applying backwards stepwise selection where only those with a *p*-value less than 0.20 were selected in the final model. Participant ID was included as the random effect to account for within-subject variability and to control for the repeated measures structure of the data. This random effect served as a proxy for time, capturing the underlying temporal structure associated with each participant's repeated measures. Each model allowed estimate the associations among changes in individuals’ anxiety and depression scores and both static and time-varying variables over the observation period. Mixed-effects models allow the inclusion of both static and time-varying predictors; uneven assessment intervals and differing number of time points across participants [58].

**Table 1:** Summary of young participant characteristics at baseline (*N* = 1936)

Participants’ characteristics (*n* = 1936)	n (%)
**Age, years, Mean(SD)**	23.3 (4.5)
**Gender**	
Female	956 (49.4)
Male	946 (48.9)
Non-binary	24 (1.2)
Other	10 (0.5)
**Age group, years**	
15–19	518 (26.8)
20–24	623 (32.2)
25–29	795 (41.0)
**State/territory**	
VIC	701 (36.2)
NSW	505 (26.1)
QLD	306 (15.8)
NT	17 (0.9)
ACT	49 (2.5)
WA	186 (9.6)
TAS	45 (2.3)
SA	124 (6.4)
I don’t wish to say	3 (0.2)
**Region**	
Metropolitan	1465 (75.7)
Inner regional	203 (10.5)
Rural or remote	83 (4.3)
Missing data	185 (9.5)
**Postcode affected by a bushfire**	
No	1802 (93.1)
Yes	134 (6.9)
**Sexual identity (LGBTQIA +)**	
No	1359 (70.2)
Yes	569 (29.4)
Missing	8 (0.4)
**Residential status in Australia**	
Citizen	1584 (81.8)
Permanent Resident	182 (9.4)
Other Temporary	135 (7.0)
I don’t wish to say	35 (1.8)
**Country of birth**	
Australia	1584 (81.8)
Other	321 (16.6)
Prefer not to say	31 (1.6)
**Aboriginal or Torres Strait Islander**	
No	1871 (96.7)
Yes	49 (2.5)
I don’t wish to say or missing	16 (0.8)
**Highest completed or enrolled level of education at baseline**	
High school or lower level	578 (29.9)
Tertiary education	1334 (68.9)
I don’t wish to say or missing	24 (1.2)
**Recruitment Source**	
Pure Profile	1117 (57.7)
Social Media	819 (42.3)
**Loneliness**	
Lower than mild loneliness	952 (49.2)
Mild loneliness or higher	951 (49.1)
Missing data	33 (1.7)

Participant characteristics at baseline among young people in Australia 2020–2021. This table features age -mean (SD), and each category distribution as n (%). Participants’ characteristics at baseline also defined as static variables

For all analyses, *p* < 0.05 was considered significant. All analyses were undertaken using Stata Statistical Software version 15 by StataCorp USA, Texas [59].

#### Sensitivity analysis

Because of differential loss to follow-up between recruitment methods (market research panel, social media), sensitivity analyses were used to investigate whether the recruitment type influenced DASS-21 anxiety and depression outcomes. Linear mixed-effects regression models similar to the primary models were used, separated by the two recruitment methods (see Additional file 2) and by states Victoria vs other states (see Additional file 3). Characteristics of participants who were more likely to remain in the study were analysed using a Chi-squared test. Additionally, we used two logistic regressions to investigate if baseline mental health scores (*n* = 1936) influenced participants continuation in the study, using continuation (yes/no) as the outcome variable and anxiety and depression as predictors in their respective models (See Additional file 4).

#### Ethics approval

Ethical approval for the project number 190/20 was granted by the Alfred Health Ethics Committee on 08 April 2020.

## Results

### Participant characteristics

A total of 2006 participants completed the baseline survey. Of these, 1936 were eligible to be included in the baseline analysis (e.g., met age criteria and completed at least 6 items of each DASS-21 anxiety and depression subscales). A total of 518 (27%) completed timepoint 2, 470 (24%) completed timepoint 3, and 397 (21%) completed timepoint 4. In total, 219 participants (11%) completed all four surveys.

At baseline, most participants were recruited from the market research panel (58%). The attrition rate from timepoints 1 to 4, was 62% for social media and 92% for panel participants. Chi-squared tests showed that participants more likely to remain in the study were female (71% vs 58% at baseline, *p* < 0.01), aged 15 to 19 years (33% vs 27% at baseline, *p* < 0.001), living in Victoria (48% vs 36% at baseline, *p* < 0.001), recruited through social media (75% vs 42% at baseline, *p* < 0.001), living with parents (51% vs 46% at baseline, *p* < 0.001), and had completed tertiary education (65% vs 69% at baseline, *p* < 0.001).

Table 1 summarises the cohort’s main demographic, socioeconomic and lifestyle characteristics at baseline. Table 2 summarises the time-varying characteristics of the cohort including changes from timepoint 1 to timepoint 4.

**Table 2:** Description of time-varying participant demographics, socioeconomic status, lifestyle and COVID-19-related-factors among young people in Australia

Participants’ time-varying variables	Pre-pandemic *n* = 1936 *†*	Apr-Sep 2020 (T1) *n* = 1,936	Jul- Dec 2020 (T2) *n* = 518	Oct 20–Mar 21(T3) *n* = 470	Apr—Aug 2021 (T4) *n* = 397
**DASS-21 Anxiety Score Mean (SD)**		9.9 (9.4)	9.9 (9.5)	9.9 (9.5)	10.2 (9.6)
**DASS-21 Depression Score Mean (SD)**		15.2 (11.5)	15.8 (11.3)	15.0 (11.8)	15.4 (11.3)
**Social media hours per day Mean (SD)**		4.1 (3.2)	3.8 (2.8)	3.6 (2.3)	3.8 (2.6)
**Living With n (%)**					
Alone		192 (9.9)	40 (7.7)	36 (7.7)	32 (8.1)
Parents		892 (46.1)	272 (52.5)	246 (52.3)	181 (45.6)
Partner		530 (27.4)	127 (24.5)	115 (24.5)	104 (26.2)
Friends/roommates		261 (13.5)	67 (13.0)	66 (14.0)	67 (16.8)
other		61 (3.1)	12 (2.3)	7 (1.5)	13 (3.3)
**In a relationship n (%)**					
No		953 (49.3)	272 (52.5)	250 (53.2)	190 (47.9)
Yes		971 (50.1)	243 (46.9)	219 (46.6)	206 (51.9)
Prefer not to say		12 (0.6)	3 (0.6)	1 (0.2)	1 (0.2)
**Student status n (%)**					
Not a current student		876 (45.2)	190 (36.7)	176 (37.5)	146 (36.8)
Going to school/university/class in person		113 (5.8)	156 (30.1)	145 (30.8)	149 (37.5)
Studying, by distance/online		845 (43.7)	154 (29.7)	124 (26.4)	84 (21.2)
Deferred, withdrawn, drop out or I don't wish to say		102 (5.3)	18 (3.5)	25 (5.3)	18 (4.5)
**In lockdown (%)**					
Yes		810 (41.8)	224 (43.2)	107 (22.8)	78 (19.7)
No		1126 (58.2)	294 (56.8)	363 (77.2)	319 (80.3)
**Current work status n (%)**					
Full-time	742 (38.3)	646 (33.4)	151 (29.1)	150 (31.9)	127 (32.0)
Part-time	288 (14.9)	278 (14.3)	55 (10.6)	46 (9.8)	59 (14.9)
Casual	469 (24.2)	302 (15.6)	119 (23.0)	134 (28.5)	120 (30.2)
Unemployed	357 (18.4)	579 (29.9)	158 (30.5)	115 (24.5)	71 (17.9)
Other	80 (4.1)	131 (6.8)	35 (6.8)	25 (5.3)	20 (5.0)
**Financial security n (%)**					
Financially secure	1575 (81.3)	1238(64.0)	395 (76.3)	353 (75.1)	289 (72.8)
Financially insecure	361 (18.7)	698 (36.0)	123 (23.7)	117 (24.9)	108 (27.2)
**Days per week having trouble to sleep n (%)**					
Zero to two days per week	764 (39.4)	711 (36.7)	254 (49.0)	257 (54.7)	208 (52.4)
Over two days per week	402(20.8)	455 (23.5)	264 (51.0)	213 (45.3)	189 (47.6)
‡ Missing	770 (39.8)	770 (39.8)	0 (0.0)	0 (0.0)	0 (0.0)

Each category distribution is presented as n (%). *Higher scores represent poorer mental health symptoms, score range [0–42], † Pre-pandemic data were collected at the baseline survey based on participants recall. Pre-pandemic questions only covered work status, financial security and days having trouble to sleep mainly for the purpose of survey length management. ‡ The substantial amount of missing data for the sleep variable at pre-pandemic and timepoint 1 is a result of a Redcap coding error that led to the exclusion of this question from the baseline survey during its initial distribution stages. “Other” work status includes self-employed, carers, and gig workers. T1,2,3,4 = Timepoint 1,2,3,4

### Changes in time-varying variables from the start of April 2020

Almost half of the sample (48%, *n* = 931) changed their employment status at least once during the observation period. There was an observed decrease in proportions of unemployment and an increase in full-time and casual work from timepoint 1 to timepoint 4. Part-time employment remained relatively stable over the observation period while ‘other’ employment categories declined (See Additional file 5).

Table 2 shows a 0.3 increase in DASS-21 anxiety mean scores from timepoint 3 (October 2020 – March 2021) to timepoint 4 (April 2021 – August 2021). Meanwhile, DASS-21 depression scores showed slight fluctuations across timepoints with the highest mean score observed in July to December 2020 (timepoint 2). This timepoint coincides with the longest lockdown in 2020 in Victoria as well as shorter lockdowns in South Australia and New South Wales (as shown in Fig. 1). By timepoint 3, rather than longer lockdowns, multiple short lockdowns were in place across multiple states, including Victoria, New South Wales, Queensland, South Australia, and Western Australia.

#### Anxiety and depression symptoms by lockdown from April 2020 –August 2021

**Fig. 2:**
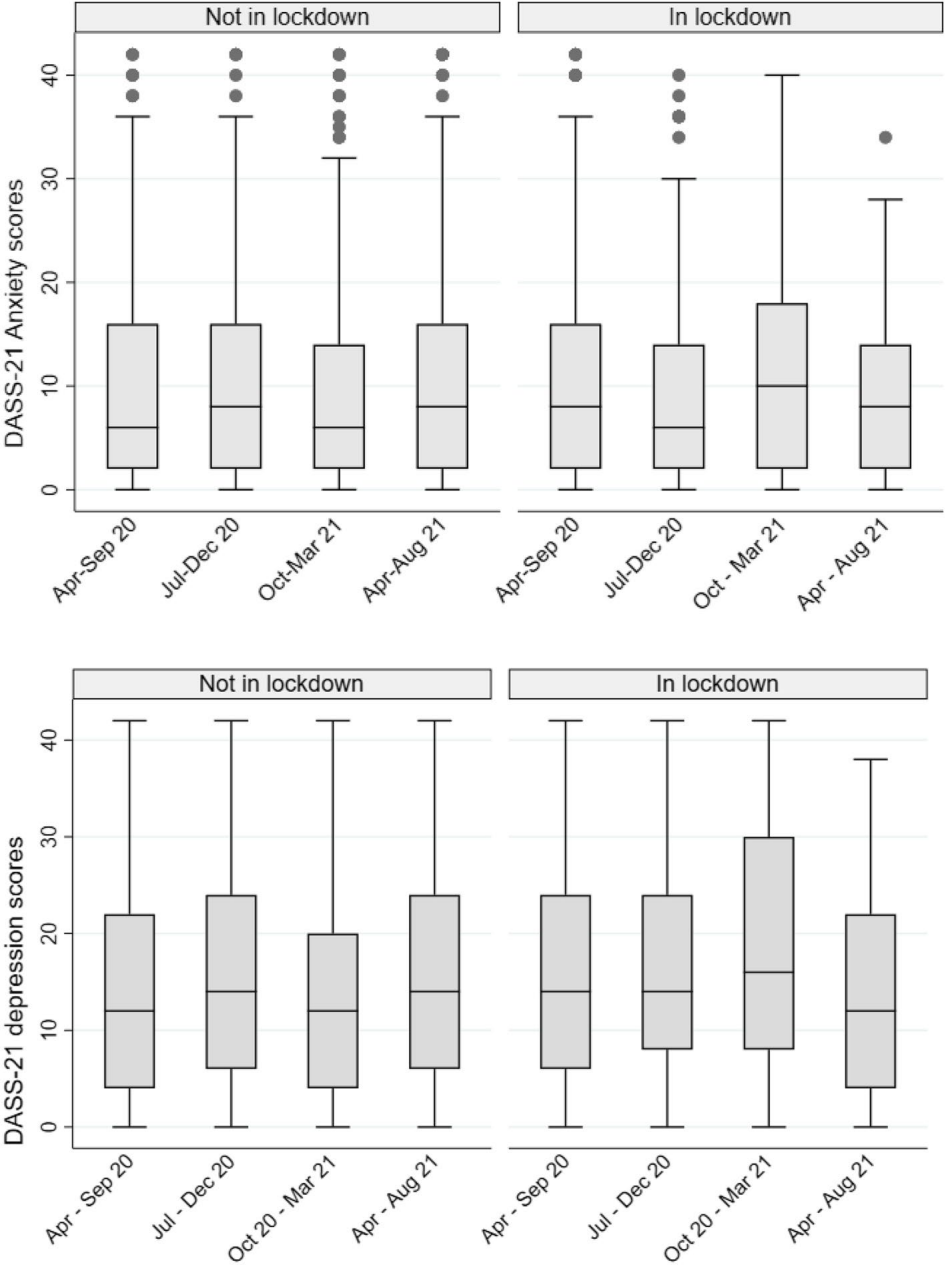
Young people’s DASS-21 anxiety and depression scores by lockdown status at each timepoint in Australia

Figure 2 shows slight changes with high variability in anxiety and depression scores over the observation period. A slight increase in both scores is observed for those in lockdown between October 2020 and March 2021 (Timepoint 3).

### Static and time-varying factors associated with changes in the severity of anxiety and depression symptoms

Table 3 shows static and time-varying factors associated with changes in anxiety and depression scores identified in the linear mixed-effects regression models.

**Table 3:** Mixed-effects regression models of anxiety and depression symptoms scores (DASS-21) among young people in Australia 2020–2021

**Factor**	Anxiety (DASS-21) *n* = 3321			Depression (DASS-21) *n* = 3321		
***Static variables***	**β-coefficient**	***p*-value**	**(95% CI)**	**β-coefficient**	***p*-value**	**(95% CI)**
**Gender (vs. male)**						
Female	0.6	0.1	(-0.2,1.4)	0.4	0.3	(-0.5,1.3)
Non-binary	5.5**	** < 0.01**	(2.4,8.6)	3.0	0.1	(-0.5,6.4)
Other	-1.7	0.5	(-6.1,2.7)	-4.0	0.1	(-8.9,0.9)
**Age group at baseline (vs. 25–29 years)**						
15–19	1.7*	** < 0.05**	(0.4,3.0)	1.2	0.1	(-0.3,2.6)
20–24	1.5**	** < 0.01**	(0.6,2.3)	0.6	0.2	(-0.3,1.6)
**Bushfire affected postcode (vs. no)**						
Yes	0.4	0.6	(-1.0,1.8)	0.4	0.6	(-1.2,2.0)
**LGBTQIA + (vs. no)**						
Yes	2.0***	** < 0.001**	(1.1,2.8)	2.3***	** < 0.001**	(1.4,3.2)
Missing	-0.9	0.6	(-4.0,2.3)	-0.5	0.8	(-4.1,3.1)
**Residential status in Australia (vs. citizen)**						
Permanent Resident	0.9	0.2	(-0.3,2.1)	0.4	0.6	(-1,1.7)
Other temporary visa	-0.1	0.9	(-1.4,1.2)	-0.5	0.5	(-2.0,1.0)
**Aboriginal or Torres Strait Islander (vs. no)**						
Yes	4.1**	** < 0.01**	(1.8,6.4)	2.2	0.1	(-0.3,4.8)
I don’t wish to say	3.3	0.1	(-0.7,7.3)	0.5	0.8	(-4.0,5.0)
**Highest completed or enrolled level of education at baseline (vs. high school)**						
Tertiary education	-0.5	0.3	(-1.5,0.5)	-1.0	0.1	(-2.1,0.1)
Missing or I don’t know	-0.8	0.6	(-4.3,2.6)	0.0(03)	1.0	(-3.9,3.9)
**Work status before the pandemic (vs. full-time)**						
Part-time	0.2	0.8	(-1.2,1.5)	-0.1	0.9	(-1.6,1.5)
Casual	-0.6	0.4	(-1.9,0.7)	-0.4	0.6	(-1.9,1.1)
Unemployed	-0.5	0.5	(-2.1,1.0)	-2.0*	** < 0.05**	(-3.8,-0.3)
Other	0.3	0.8	(-1.9,2.4)	-1.0	0.4	(-3.3,1.4)
**Financial security before the pandemic (vs.financially secure)**						
Financially insecure	2.9***	** < 0.001**	(1.9,3.9)	2.5***	** < 0.001**	(1.4,3.6)
**Recruitment approach (vs. research market panel)**						
Social Media	0.1	0.8	(-0.8,1.0)	1.0	0.1	(0,2.1.0)
**Loneliness (vs. mild loneliness or lower)**						
Moderate loneliness or higher	4.2***	** < 0.001**	(3.4,4.9)	7.8***	** < 0.001**	(7.0,8.7)
Missing data	5.1***	< 0.001	(2.3,7.9)	5.7***	< 0.001	(2.5,8.8)
***Time-varying variables***						
**Hours spent on social media per day**	0.3***	** < 0.001**	(0.2,0.4)	0.4***	** < 0.001**	(0.2,0.5)
**Living with (vs. alone)**						
Parents	-2.3***	** < 0.001**	(-3.5,-1.2)	-1.5*	** < 0.05**	(-2.8,-0.1)
Partner	-2.8***	** < 0.001**	(-4.0,-1.5)	-2.3**	** < 0.01**	(-3.7,-0.8)
Friends/roommates	-1.5*	** < 0.05**	(-2.7,-0.2)	-0.6	0.4	(-2.0,0.8)
Other	-1.8	0.1	(-3.6,0.1)	-1.2	0.3	(-3.3,0.9)
**In a relationship (vs. no)**						
Yes	1.2**	** < 0.01**	(0.4,1.9)	0.7	0.1	(-0.1,1.6)
Prefer not to say	0.0(04)	0.9	(-3.6,3.6)	-1.5	0.5	(-5.7,2.6)
**Student status (vs. not a current student)**						
Going to school/university/class in person	2.1***	** < 0.001**	(1.1,3.0)	0.5	0.4	(-0.6,1.6)
Studying, by distance/online	0.5	0.2	(-0.2,1.3)	0.0	0.9	(-0.8,0.9)
Deferred/withdrawn/dropped studies	2.2**	** < 0.01**	(0.9,3.5)	2.2**	** < 0.01**	(0.8,3.7)
**Current work status (vs. full-time)**						
Part-time	-0.7	0.2	(-1.9,0.4)	0.1	0.9	(-1.2,1.4)
Casual	-1.2	0.0(05)	(-2.3,0.0)	0.8	0.2	(-0.5,2.1)
Unemployed	-1.4*	** < 0.05**	(-2.6,-0.2)	2.1**	** < 0.01**	(0.7,3.4)
Other	-0.2	0.8	(-1.7,1.2)	2.6**	** < 0.01**	(1.0,4.3)
**Financial security when taking the survey (vs. Financially secure)**						
Financially insecure	1.4***	** < 0.001**	(0.8,2.1)	1.8***	** < 0.001**	(1.0,2.5)
**In lockdown (vs. no)**						
Yes	0.2	0.4	(-0.3,0.8)	1.0**	** < 0.01**	(0.4,1.6)
**Days per week having trouble to sleep (vs. zero to two days per week)**						
Over two days per week	3.0***	** < 0.001**	(2.4,3.6)	4.3***	** < 0.001**	(3.6,5)
Missing	0.6	0.1	(-0.1,1.3)	2.0***	< 0.001	(1.2,2.9)
**Constant**	4.5***	** < 0.001**	(3.0,6.1)	3.6***	** < 0.001**	(2.5,4.7)
**Random effects**	**Estimate**	**SE**	**95% CI**	**Estimate**	**SE**	**95% CI**
**Participant Id (constant)**	41.1	2.1	(37.2,45.4)	49.0	2.6	(44.2,54.3)
**var (residual)**	26.6	1.0	(24.7,28.7)	37.1	1.4	(34.4,39.9)

This table presents the name of the variables as factor (vs. reference group)

^*^p < .05, ** *p* < .01, *** *p* < .001; *CI* Confidence interval; Tests used: Likelihood Ratio Test anxiety = -11,336.4, Likelihood Ratio Test depression = -11,785.8, Wald Chi-Squared Test depression = 1240.1 and Wald Chi-Squared Test Anxiety = 738.2; “Other” work status includes self-employed, carers, and gig workers. *SE Standard error

#### Anxiety

##### Static factors

Identifying as non-binary gender, LGBTQIA + or Aboriginal or Torres Strait Islander, experiencing financial insecurity before the pandemic, and experiencing moderate to severe feelings of loneliness at baseline were associated with increased anxiety scores. Being aged 25–29 years compared to 15–19 and 20–24 was associated with a decrease in anxiety scores from timepoint 1 to 4.

##### Time-varying factors

Experiencing financial insecurity, spending more time on social media per day, being in a relationship, studying in person, having deferred, withdrawn, or dropped studies, and having sleeping problems two or more days per week were factors associated with increased anxiety scores. Transitioning between full-time employment and unemployment, and changes in living circumstances, including living with parents, a partner, friends, or roommates compared to living alone, contributed to lower anxiety scores.

#### Depression

##### Static factors

Identifying as LGBTQIA + , experiencing financial insecurity before the pandemic, and experiencing moderate to severe feelings of loneliness at baseline were associated with increased depression scores. Being unemployed before the pandemic was associated with a decrease in depression scores when compared to being full-time employed before the pandemic.

##### Time-varying factors

Experiencing financial insecurity, spending more time on social media per day, having deferred, withdrawn, or dropped studies, transitioning between full-time employment and unemployment or holding an alternative job type, being in lockdown, and reporting sleeping problems over two days per week were factors associated with increased depression scores. Compared to living alone, transitioning to living with parents or a partner was associated to a decrease in depression scores over time.

### Sensitivity analysis

The mixed-effect regression models adjusting for recruitment type (See Additional file 2), yielded similar results to primary analysis, with some slight differences. In the Pure Profile sample, a significant increase in anxiety scores was observed only among those aged 20–24 and who were under lockdown, while student status and employment status were no longer significant compared to primary analysis. In the social media cohort, residency status became significant, with permanent residents showing increased anxiety scores. Additionally, changes in depression scores differed in the social media cohort, with females and those with higher education showing significant effects, whereas living alone was no longer significant. After adjusting for states (VIC vs. others), many results shifted to non-significance. This may be because 70% of those in lockdown were from Victoria, explaining the non-significant lockdown effect in other states. Additionally, bushfire effects became significant in the VIC model, though only 3 participants from Victoria were affected compared to 127 in NSW. Additionally, the two logistic regressions assessing whether dropout rates were associated with DASS-21 baseline scores showed no significant differences in mental health between those who dropped out of the study and those who continued (see Additional file 4).

## Discussion

We conducted longitudinal analyses to investigate factors associated with changes in the severity of anxiety and depression symptoms among young people living in Australia amidst the COVID-19 pandemic from April 2020 to August 2021.Compared to a study conducted with young Australians aged 18–24 during April 2020, DASS-21 anxiety and depression mean scores were similar to those without a mental health diagnosis and were lower than those with an existing mental health diagnosis [34]. Our findings showed significant variability in anxiety and depression severity symptoms between participants.

Longitudinal analyses identified several key risk factors associated with increases in anxiety and depression symptoms including LGBTQIA + identity, financial insecurity both before and during the pandemic, higher levels of loneliness, withdrawal or deferral of studies, spending more time on social media, and difficulties to sleep. Risk factors for only depression symptoms include unemployment during COVID-19 pandemic and being in lockdown. The study also identified several protective factors including pre-COVID-19 unemployment associated with a decrease in depression symptoms, while older age, unemployment during the pandemic, and living with someone were all associated with reduced anxiety symptoms. Some of these factors are common risk factors for higher levels of mental health symptoms identified before COVID-19 [2, 60]. However, these findings suggest that these factors may also impact the longitudinal course of symptoms, contributing to either their improvement or exacerbation over time. These findings suggest the need for interventions to support the mental health of young Australians during the recovery from the COVID-19 pandemic and in preparation for future pandemics.

This research reveals significant changes in anxiety and depression symptom severity among young Australians at the individual level, even though the mean scores remained relatively stable across four distinct time points. This divergence in individual experiences underscores the significant role of longitudinal data in identifying factors linked to shifts in DASS-21 anxiety and depression scores.

Our results are consistent with other longitudinal studies conducted globally and in Australia that indicated a rise in anxiety and/or depression symptoms in young people during the COVID-19 pandemic [15, 17, 20, 21]. However, our research also highlights the critical role of individual differences and factors in shaping young people’s psychological response to the pandemic and related disruptions.

This study’s findings align with literature indicating that various factors were associated with higher levels of anxiety and/or depression among young people, including being aged 18–24 years [17, 25, 34], identifying as LGBTQIA + [30, 31], identifying as nonbinary [5, 21] increased social media use [24] and having sleep problems [15]. We did not find any evidence to support an association between the primary outcomes and living in an area recently affected by bushfires [44]. Our results contribute to existing research by highlighting longitudinal factors influencing the mental health of young people aged 15 to 29 in Australia during the COVID-19 pandemic.

This study found that Aboriginal and Torres Strait Islander youth were more likely than other young people to experience a significant increase in the severity of anxiety symptoms over time. This is not surprising, given that prior to the COVID-19 pandemic, Aboriginal and Torres Strait Islander adolescents reported significantly higher rates of psychological distress and depression when compared to non-Indigenous adolescents [61]. We recognise that past and present personal, family and community experiences of trauma (driven by the effects of colonisation) underpin these statistics [61] and that during the COVID-19 pandemic limited access to culturally sensitive and safe mental health services to meet their needs is likely to have contributed to this increase [62]. Additionally to this Aboriginal and Torres strait Islander young people anxiety symptomology may not be measured accurately by the DASS-21, given that anxiety sits within a holistic experience of wellbeing for many Aboriginal and Torres Strait Islander youth [63]. To appropriately interpret these results, we recommend a self-determined response that is developed and led by Aboriginal and Torres Strait Islander communities with participation from Aboriginal and Torres Strait Islander young people to understand the adequacy and what underpins these statistics.

### Demographic correlates

Our study conflicts with previous literature [13, 20, 28, 29] that identified being female as a significant risk factor for anxiety and depression symptoms worsening. We found that being female was not a significant predictor of the impact of the COVID-19 pandemic on the mental health of young adults in our sample. These results indicate that, irrespective of their initial mental health status, males and females encountered comparable levels of mental health changes during the pandemic. However, non-binary people were more prone to experience worsening anxiety symptoms over the observation period, in line with existing literature [5, 21].

Additionally, our research found that participants who transitioned between full-time employment and unemployment, self-employment, caregiving roles, or gig work during the observation period, were more likely to experience worsening depression symptoms over time. This may be due to increased financial burden or loss of some benefits of employment such as identity and purposeful used of time [64]. Conversely, participants who experienced changes in employment status, such as transitioning between full-time employment and unemployment, were less likely to experience worsening anxiety symptoms. This may be attributable to Australian government financial support programs mitigating some of the stress associated with unemployment. Nonetheless, financial insecurity was identified as a risk factor for worsening anxiety and depression symptoms among young people in our sample despite the extension of government financial support programs [65]. This may be due to not all young people meeting the criteria for accessing government financial support.

### Lockdown correlates

Our study findings indicate that young people who were in lockdown during the observation period experienced an increase in the severity of depression symptoms. This aligns with the findings of a systematic review in which depressive symptoms but not anxiety symptoms were higher during periods of social restrictions [1, 26]. Our findings support previous research, which showed that young Australians (18–24 years old) living with their parents or partners were less likely to experience severe psychological distress than those living alone [13, 16]. Lockdown restrictions may not have impacted on anxiety in the same way. Considering the different facets of anxiety, a reduction in socialisation may have led to a decrease in social anxiety, while social distancing may have reduced fear about the risk of COVID-19 infection [37]. The high levels of loneliness reported in our study are concerning as loneliness is a risk factor for anxiety and depression disorders and suicidal ideation [66]. To address this issue, we recommend implementing more inclusive public health restrictions. The restrictions in 2020–2021 often catered to normative family and relationship structures, neglecting the needs of individuals who live alone or do not conform to these structures. We suggest consulting with those living alone or in non-heteronormative relationships to generate ideas on how to make more inclusive public health guidelines.

### Limitations

This study had some limitations. The use of a non-probability sampling method and a high attrition rate may constrain the generalisability of the findings. While sensitivity analyses suggest that findings remain comprehensive across different recruitment methods (e.g., social media and research market panels), future studies should attempt to recruit a more representative sample and investigate ways to improve retention. The high attrition rate among panel participants may have been influenced by the change in reimbursement methods for Pure Profile participants following survey 1, potentially impacting their motivation to continue participating [67]. If resourced sufficiently, researchers could consider contacting participants via phone or text message to keep them informed of the project’s progress and provide an alternative method to complete the survey such as a phone interview [68]. This approach could potentially enhance participant retention rates and improve the study's overall representativeness. This said, there are considerable logistical challenges in implementing a longitudinal study when a pandemic begins, including securing funding that allow for more intensive contact. Further, it is understandable that retention is constrained during times of widespread hardship and uncertainty.

This study was unable to assess some potential risk factors for anxiety and depression symptoms such as history of mental illness or perceived risk of COVID-19 infection or actual infection. Previous research has shown, young individuals diagnosed or suspected of being infected with COVID-19 and people with pre-existing mental illness have reported higher anxiety and depression scores [17, 21, 34].

Because it was implemented in response to the pandemic, this study lacked pre-pandemic baseline measures. All study data related to pre-pandemic experiences is based on participants’ recall. Furthermore, the most recent publicly accessible national mental health data concerning Australian youth, prior to the 2020 pandemic, dates back to 2007 [69]. As such, data may not capture all relevant factors or situations. Another limitation is not performing imputation for missing data as part of the sensitivity analysis, considering the exploratory nature of the study and the risk of introducing additional assumptions and potential bias. As a result, the findings should be interpreted with caution. Additionally, it is worth considering that the reliability of the DASS-21 scale for people under 17 is ambiguous [70–72], indicating the need for a different scale with higher reliability for young people in this age group such as DASS-Y developed in 2022 [73].

Moreover, this study did not disaggregate the direction of the associations found between various factors and anxiety and depression symptoms nor reported their temporal and causal structure. For instance, the association between change in employment and change in anxiety scores could be due to a combination of factors, such as people who become employed reporting decreased anxiety scores and people who become unemployed reporting increased anxiety scores. While specifying these correlations would provide more detailed information, it could also spread the data more sparsely, limiting statistical power and potentially reducing the study's ability to draw meaningful conclusions. Therefore, future studies could benefit from exploring the direction and causal structure of associations more thoroughly, while also considering the potential impact on statistical power. Lastly, to clearly differentiate between short-term fluctuations from more stable changes in symptom severity, further investigation is warranted. One alternative could be to incorporate daily or weekly tracking of symptoms.

### Strengths

This study benefited from a unique opportunity to explore the effects of prolonged lockdowns on anxiety and depression symptoms among young Australians. This was possible because participants' lockdown status after the first Australian national lockdown on March 30 varied by state, allowing for a natural experiment. This study also provides an understanding of what could exacerbate or mitigate young people’s mental health issues during public health emergencies. This dual focus could guide interventions to enhance protective factors and mitigate risks. Taken together, these findings advance our understanding of the complex interplay between the pandemic, public health policies, and mental health outcomes.

### Implications for policy and practice

Public health strategies to protect mental health should target groups most vulnerable to the pandemic's impact to reduce anxiety and depression symptoms during the COVID-19 pandemic recovery. Additionally, it is crucial to attain a deeper understanding of the factors associated with improved mental health symptoms during such crises to inform prevention and support initiatives.

Our study reinforced the importance of employment and education as a protective factor for young people’s mental health [74], including during pandemics. In the ongoing response and recovery efforts during the COVID-19 pandemic, it remains imperative to prioritise providing young people with more accessible training to cultivate transferable skills, creating stable employment opportunities, and upholding existing jobs. To mitigate mental health risks among young people, it is also vital to target financial support programs at those facing financial hardship, regardless of their employment status. Expanding eligibility criteria to encompass financially vulnerable young individuals can furnish essential support to this demographic, potentially fostering positive impacts on their mental well-being.

## Conclusion

Adding to existing work, our cohort study reveals significant shifts in the mental health of young Australians during the COVID-19 pandemic from 2020 to 2021. We found young people who are younger, LGBTQIA + , non-binary gender, experiencing financial insecurity, facing lockdowns, dealing with unstable employment, enduring loneliness, spending more time on social media, and living alone tended to experience worsened mental health. This highlights the necessity for targeted interventions and ongoing support for these subgroups. To enhance future pandemic and public health crises responses, we suggest more inclusive guidelines that involve young people in their development and implementation ensuring that their unique perspectives and needs are adequately considered. Finally, future longitudinal studies should implement strategies to decrease attrition among young people. Such studies would help identify the critical time periods when young people are most at risk and provide deeper insights into their evolving health needs during public health crises and their recovery.

## Supplementary Information

Supplementary Material 1.

Supplementary Material 2.

Supplementary Material 3.

Supplementary Material 4.

Supplementary Material 5.

## Data Availability

The data that support this study will be shared upon reasonable request to the corresponding author.

## Abbreviations

REDCap: Research Electronic Data Capture
LGBTQIA +: Lesbian, gay, bisexual, transgender, intersex, queer/questioning, asexual
COVID-19: Coronavirus disease 2019
USA: United States of America
NSW: New South Wales
VIC: Victoria
TAS: Tasmania
QLD: Queensland
WA: West Australia
SA: South Australia
NT: Northern Territory
ACT: Australian Capital Territory

## Data availability

No datasets were generated or analysed during the current study.
