# Supplementary material for: Coping with COVID-19: a prospective cohort study on young Australians' anxiety and depression symptoms from 2020–2021
Source: Arch Public Health. 2024 Sep 26;82:166. doi: 10.1186/s13690-024-01397-z (PMC11426065; doi:10.1186/s13690-024-01397-z)
Supplement: Supplementary file 1 — Supplementary Material 1. [file 13690_2024_1397_MOESM1_ESM.docx]

**Additional File 1:** Lockdown and bushfire dates per Australian state/territory from April 2020 to August 2021

| State | Lockdown start | Lockdown end | Bushfires | Bushfire Included Postcodes | Time  points |
| --- | --- | --- | --- | --- | --- |
| VIC | 30/03/2020 | 12/05/2020 | January 2020 | 3691 3697 3698 3699 3737 3738 3739 3740 3741 3744 3862 3898 3699 3701 3707 3851 3864 3865 3875 3878 3880 3882 3885 3886 3887 3888 3889 3890 3891 3892 3893 3895 3896 3898 3900 3902 3903 3904 3909 3695 3700 3704 3705 3708 3709 3690 3694 | 1 |
|  | 30/06/2020 | 27/10/2020 |  |  | 1 & 2 |
|  | 13/02/2021 | 17/02/2021 |  |  | 3 |
|  | 28/05/2021 | 10/06/2021 |  |  | 4 |
|  | 15/07/2021 | 27/07/2021 |  |  | 4 |
|  | 5/08/2021 | 30/08/2021 |  |  | 4 |
|  |  |  |  |  |  |
| SA | 29/03/2020 | 10/05/2020 | November-December 2019 | 5052 5072 5073 5076 5114 5125 5131 5132 5133 5134 5136 5137 5138 5139 5140 5141 5142 5144  5151 5152 5153 5154 5155 5156 5157 5231 5232 5233 5234 5235 5240 5241 5242 5243 5244 5245 5252 5351 5110 5112 5113 5114 5115 5116 5117 5118 5120 5121  5220 5221 5222 5223 5264 5271 5273 5275 5606 5607 5630 5631 5632 5153 5155 5157 5201 5243 5244 5245 5250 5251 5252 5254 5552 5558 5570 5571 5572 5573  5575 5576 5577 5580 5581 5582 5583 | 1 |
|  | 19/11/2020 | 21/11/2020 |  |  | 2 |
|  | 20/07/2021 | 27/07/2021 |  |  | 4 |
|  |  |  |  |  |  |
| QLD | 29/03/2020 | 27/05/2020 | September – November 2019 | 4670 4674 4676 4677 4678 4680 4694 4695 4697 4741 4700 4701 4702 4703 4704 4705 4706 4710 4711 4741 4562 4563 4565 4566 4567 4568 4569 4571 4573 4306 4311 4312 4313 4314 4342 4346 4514 4515 4314 4350 4352 4353 4354 4355 4356 4357 4358 4359  4360 4361 4363 4364 4365 4400 4401 4402 4403 4404 4405 4407 4614 | 1 |
|  | 8/01/2021 | 11/01/2021 |  |  | 3 |
|  | 29/03/2021 | 1/04/2021 |  |  | 3 & 4 |
|  | 29/06/2021 | 02/07/2021 |  |  | 4 |
|  | 31/07/2021 | 11/08/2021 |  |  | 4 |
|  |  |  |  |  |  |
| WA | 16/03/2020 | 27/04/2020 |  |  | 1 |
|  | 31/01/2021 | 5/02/2021 |  |  | 3 |
|  | 24/04/2021 | 26/04/2021 |  |  | 4 |
|  | 28/06/2021 | 2/07/2021 |  |  | 4 |
|  |  |  |  |  |  |
| NSW | 31/03/2020 | 15/05/2020 | September November 2019 | 2350 2351 2359 2360 2365 2369 2370 2440 2453 2546 2548 2549 2550 2551 2631 2632 2448 2449 2453 2454 2455 2758 2773 2774 2776 2777 2778 2779 2780 2782 2783 2784 2785 2786 2787 2083 2250 2251 2256 2257 2258 2259 2260 2261 2262 2263 2775 2250 2320 2321 2322 2323 2325 2326 2327 2334 2335 2365 2370 2450 2453 2456 2460 2462 2463 2464 2465 2466 2469 2472 2450 2452 2454 2456 2460 2536 2537 2545 2546 2550 2622 2365 2370 2371 2579 2580 2581 2622 2640 2642 2644 2646 2650 2652 2658 2659 2660 2330 2753 2754 2755 2756 2757 2758 2765 2775 2360 2361 2365 2369 2370 2371 2403 2410 4385 2431 2440 2441 2469 2470 2474 2475 2476 2480 2470 2471 2472 2477 2480 2785 2786 2787 2790 2795 2845 2846 2847 2848 2849 2850 2312 2324 2337 2415 2422 2423 2424 2425 2426 2427 2428 2429 2430 2443 2795 2844 2848 2849 2850 2852 2441 2447 24482449 2454 2580 2787 2790 2795 2429 2439 2440 2441 2443 2444 2445 2446 2580 2581 2619 2620 2621 2622 2623 2469 2470 2471 2472 2473 2480 2534 2535 2536 2538 2539 2540 2541 2577 2622 2320 2325 2330 2331 2333 2335 2546 2551 2620 2621 2622 2625 2626 2627 2628 2629 2630 2631 2632 2633 2611 2627 2629 2640 2642 2649 2652 2653 2720 2722 2729 2730 3707 2371 2372 2469 2475 2476 4375 4377 4380 4383 2483 2484 2485 2486 2487 2488 2489 2490 2579 2580 2581 2582 2583 2787 2350 2354 2358 2359 2365 2354 2571 2574 2575 2576 2577 2578 2579 2580 2508 2560 2568 2569 2570 2571 2572 2573 2574 2745 2752 2787 | 1 |
|  | 19/12/2020 | 9/01/2021 |  |  | 2 & 3 |
|  | 23/06/2021 | 30/08/2021 |  |  | 4 |
|  |  |  |  |  |  |
| NT | 14/04/2020 | 30/05/2020 |  |  | 1 |
|  | 27/06/2021 | 2/07/2021 |  |  | 4 |
|  | 16/08/2021 | 19/08/2021 |  |  | 4 |
|  |  |  |  |  |  |
| ACT | 23/03/2020 | 1/05/2020 | January 2020 | 2611 | 1 |
|  | 12/08/2021 | 30/08/2021 |  |  | 4 |
|  |  |  |  |  |  |
| TAS | 30/03/2020 | 11/05/2020 |  | 7212 7213 7214 7215 7216 7264 7030 7140 7304 7017 7026 7027 7030 7119 7120 7140 7172 7190 | 1 |
| Note: lockdown end date is listed as August 31, but this indicates when data collection ceased rather than the actual conclusion of the lockdowns. | | | | | |
