# Supplementary material for: Coping with COVID-19: a prospective cohort study on young Australians' anxiety and depression symptoms from 2020–2021
Source: Arch Public Health. 2024 Sep 26;82:166. doi: 10.1186/s13690-024-01397-z (PMC11426065; doi:10.1186/s13690-024-01397-z)
Supplement: Supplementary file 2 — Supplementary Material 2. [file 13690_2024_1397_MOESM2_ESM.docx]

**Additional Material**

| Additional file 2. Mixed-effects of factors associated with DASS-21 scores stratified by recruitment type social media and research market panel among young people in Australia 2020-2021 | | | | | | | | | | | | | | | | | |
| --- | --- | --- | --- | --- | --- | --- | --- | --- | --- | --- | --- | --- | --- | --- | --- | --- | --- |
| Potential Risk factor | | **Anxiety (DASS-21)- Research Market Panel (n=1455)** | | | **Anxiety (DASS-21)- social media (n=1866)** | | | **Depression (DASS-21)- Research Market Panel (n=1455)** | | | | | | **Depression (DASS-21)- social media (n=1866)** | | | |
|  |  | **β** | **P** | **(95% CI)** | **β** | **P** | **(95% CI)** | **β** | **P** | | | | **(95% CI)** | **β** | **P** | | **(95% CI)** |
| Gender (vs. male) | |  |  |  |  |  |  |  | |  | | |  |  |  | |  |
|  | Female | -0.2 | 0.7 | (-1.2,0.8) | 1.9** | **<0.01** | (0.6,3.2) | -0.3 | | 0.6 | | | (-1.4,0.8) | 1.7* | **<0.05** | | (0.2,3.2) |
|  | Non-binary | 9.1 | 0.1 | (-2.2,20.4) | 5.7** | **<0.01** | (2.2,9.1) | -4.9 | | 0.5 | | | (-17.5,7.8) | 3.7 | 0.1 | | (-0.2,7.5) |
|  | Other | -8.8* | **<0.05** | (-17.4,-0.3) | 1.7 | 0.5 | (-3.7,7.1) | -12.5* | | **<0.05** | | | (-22,-3.0) | -0.5 | 0.9 | | (-6.5,5.4) |
| Age group (vs. 25-29 years) | |  |  |  |  |  |  |  | |  | | |  |  |  | |  |
|  | 15-19 | 1.0 | 0.2 | (-0.7,2.7) | 1.0 | 0.4 | (-1.2,3.3) | 0.4 | | 0.7 | | | (-1.5,2.3) | -0.1 | 1.0 | | (-2.6,2.4) |
|  | 20-24 | 1.7** | **<0.01** | (0.7,2.7) | 1.1 | 0.1 | (-0.3,2.6) | 0.8 | 0.2 | | | | (-0.4,1.9) | 0.2 | 0.8 | | (-1.5,1.8) |
| Bushfire affected postcode (vs. no) | |  |  |  |  |  |  |  |  | | | |  |  |  | |  |
|  | Yes | 0.7 | 0.4 | (-1.0,2.4) | 0.0 | 1.0 | (-2.4,2.4) | 0.0(01) | 1.0 | | | | (-1.9,1.9) | 0.9 | 0.5 | | (-1.8,3.6) |
| LGBTQIA+ (vs. no) | |  |  |  |  |  |  |  |  | | | |  |  |  | |  |
|  | Yes | 1.0 | 0.1 | (-0.2,2.2) | 2.5*** | **<0.001** | (1.4,3.7) | 0.7 | 0.3 | | | | (-0.6,2.0) | 3.4*** | **<0.001** | | (2.2,4.7) |
|  | Missing | 0.6 | 0.8 | (-4.9,6.1) | -1.6 | 0.4 | (-5.5,2.3) | 1.4 | 0.7 | | | | (-4.8,7.7) | -0.3 | 0.9 | | (-4.8,4.1) |
| Residential status in Australia (vs. citizen) | | | |  |  |  |  |  |  | | | |  |  |  | |  |
|  | Permanent Resident | 0.0 | 1.0 | (-1.6,1.5) | 2.0* | **<0.05** | (0.1,3.9) | 0.3 | 0.8 | | | | (-1.5,2.0) | 0.8 | 0.5 | | (-1.3,2.8) |
|  | Other Temporary visa | -0.3 | 0.7 | (-1.9,1.2) | 0.0(04) | 1.0 | (-2.5,2.6) | -0.4 | 0.6 | | | | (-2.1,1.3) | -1.0 | 0.5 | | (-3.8,1.9) |
| Aboriginal or Torres Strait Islander (vs. no) | | | |  |  |  |  |  |  | | | |  |  |  | |  |
|  | Yes | 4.5** | **<0.01** | (1.9,7.2) | 2.6 | 0.2 | (-1.8,7.1) | 3.9** | **<0.01** | | | | (1,6.9.0) | -0.8 | 0.7 | | (-5.8,4.1) |
|  | I don’t wish to say | 4.6* | **<0.05** | (0.2,8.9) | 0.2 | 1.0 | (-8.5,8.9) | 2.7 | 0.3 | | | | (-2.2,7.6) | -2.5 | 0.6 | | (-12.2,7.1) |
| Highest completed or enrolled level of education at baseline (vs. high school) | | | | | |  |  |  |  | | | |  |  |  | |  |
|  | Tertiary education | 0.7 | 0.3 | (-0.5,2.0) | -1.6 | 0.1 | (-3.3,0.2) | 0.4 | 0.6 | | | | (-1.0,1.8) | -2.6* | **<0.05** | | (-4.5,-0.6) |
|  | Missing or I don't know | 0.8 | 0.7 | (-3.1,4.8) | -2.8 | 0.4 | (-10,4.4) | 0.5 | 0.8 | | | | (-3.9,4.9) | 2.8 | 0.5 | | (-5.2,10.8) |
| Work status before the pandemic (vs. full-time) | | | | |  |  |  |  |  | | | |  |  |  | |  |
|  | Part time | 0.4 | 0.6 | (-1.3,2.1) | 0.2 | 0.8 | (-2,2.5) | -0.3 | 0.7 | | | | (-2.2,1.6) | 0.8 | 0.6 | | (-1.8,3.3) |
|  | Casual | -0.2 | 0.8 | (-2.0,1.6) | -1.0 | 0.4 | (-3,1.1) | 1.0 | 0.3 | | | | (-1.0,3.0) | -1.6 | 0.2 | | (-3.9,0.7) |
|  | Unemployed | -0.2 | 0.9 | (-2.2,1.9) | -0.9 | 0.5 | (-3.2,1.4) | -1.6 | 0.2 | | | | (-4.0,0.7) | -2.8* | **<0.05** | | (-5.4,-0.2) |
|  | Other | 1.8 | 0.2 | (-0.9,4.5) | -0.7 | 0.7 | (-4.4,3) | 2.7 | 0.1 | | | | (-0.3,5.7) | -2.2 | 0.3 | | (-6.3,1.9) |
| Financially security before the pandemic (vs. secure) | | | | |  |  |  |  |  | | | |  |  |  | |  |
|  | Financially insecure | 2.7*** | **<0.001** | (1.4,4.0) | 3.3*** | **<0.001** | (1.6,5.0) | 1.7 * | **<0.05** | | | | (0.3,3.1) | 3.7*** | **<0.001** | | (1.9,5.6) |
| Loneliness (vs. less than mild loneliness) | | |  |  |  |  |  |  |  | | | |  |  |  | |  |
|  | Mild loneliness or higher | 4.2*** | **<0.001** | (3.2,5.1) | 4.4*** | **<0.001** | (3.2,5.5) | 8.0*** | **<0.001** | | | | (6.9,9.0) | 7.6*** | **<0.001** | | (6.3,8.9) |
|  | Missing data | 5.1* | **<0.05** | (1.8,8.4) | 4.9 | 0.1 | (0,9.8) | 5.7** | **<0.01** | | | | (2,9.4.0) | 5.4 | 0.1 | | (0.0,10.9) |
| Hours spent on social media per day | | 0.4*** | **<0.001** | (0.3,0.6) | 0.2** | **<0.01** | (0.1,0.4) | 0.3** | **<0.01** | | | | (0.1,0.4) | 0.4*** | **<0.001** | | (0.2,0.5) |
| Living with (vs. alone) | |  |  |  |  |  |  |  |  | | | |  |  |  | |  |
|  | Parents | -3.4*** | **<0.001** | (-4.9,-1.9) | -0.9 | 0.4 | (-2.7,1) | -3.0*** | **<0.001** | | | | (-4.7,-1.3) | 0.3 | 0.8 | | (-1.8,2.4) |
|  | Partner | -3.4*** | **<0.001** | (-5.1,-1.6) | **-1.9** | **<0.05** | (-3.8,0) | -3.5*** | **<0.001** | | | | (-5.4,-1.5) | -1.1 | 0.3 | | (-3.3,1.1) |
|  | Friends/roommates | -2.3** | **<0.01** | (-3.9,-0.6) | -0.1 | 0.9 | (-2,1.7) | -1.6* | **<0.05** | | | | (-3.5,0.3) | 0.8 | 0.5 | | (-1.3,2.9) |
|  | other | -4.0** | **<0.01** | (-6.5,-1.5) | 0.6 | 0.7 | (-2.1,3.2) | -4.1*** | **<0.001** | | | | (-6.9,-1.3) | 1.2 | 0.4 | | (-1.8,4.3) |
| In a relationship (vs. no) | | |  |  |  |  |  |  |  | | | |  |  |  | |  |
|  | Yes | 1.2* | **<0.05** | (0.01,2.4) | 1.1* | **<0.05** | (0.1,2.0) | 1.0 | 0.1 | | | | (-0.3,2.4) | 0.5 | 0.4 | | (-0.6,1.6) |
|  | Prefer not to say | 0.4 | 0.9 | (-5.2,6.0) | 0.0(09) | 1.0 | (-4.7,4.8) | 0.0(04) | 1.0 | | | | (-6.2,6.3) | -1.3 | 0.6 | | (-6.9,4.2) |
| Student status (vs. not a current student) | | | |  |  |  |  |  |  | | | |  |  |  | |  |
|  | Going to school/university/class in person | 2.5* | **<0.05** | (1.0,4.0) | 2.0** | **<0.01** | (0.6,3.3) | 0.9 | 0.3 | | | | (-0.8,2.6) | 0.3 | 0.7 | | (-1.2,1.9) |
|  | Studying, by distance/online | -0.3 | 0.6 | (-1.3,0.8) | 0.9 | 0.1 | (-0.2,1.9) | -0.7 | 0.2 | | | | (-1.9,0.4) | 0.2 | 0.8 | | (-1.1,1.4) |
|  | Deferred, withdrawn, drop out or I don't wish to say | 1.2 | 0.2 | (-0.7,3.0) | 2.9** | **<0.01** | (1.1,4.6) | 1.3 | 0.2 | | | | (-0.8,3.4) | 2.9** | **<0.01** | | (0.9,5.0) |
| Current work status (vs. full-time) | | | |  |  |  |  |  |  | | | |  |  |  | |  |
|  | Part-time | -1.3 | 0.1 | (-2.9,0.3) | 0.0 | 1.0 | (-1.6,1.6) | -0.3 | 0.8 | | | | (-2.0,1.5) | 0.8 | 0.4 | | (-1.1,2.7) |
|  | Casual | -0.8 | 0.3 | (-2.5,0.8) | -1.0 | 0.2 | (-2.6,0.6) | 0.2 | 0.8 | | | | (-1.6,2.1) | 1.8 | 0.1 | | (0,3.7) |
|  | Unemployed | -1.6 | 0.1 | (-3.2,0) | -0.9 | 0.3 | (-2.6,0.7) | 1.8 | 0.1 | | | | (0.0,3.6) | 3.1** | **<0.01** | | (1.2,5.1) |
|  | Other | -1.1 | 0.3 | (-3.1,1) | 0.4 | 0.7 | (-1.6,2.5) | -1.3 | 0.3 | | | | (-3.7,1.0) | 5.9*** | **<0.001** | | (3.5,8.3) |
| Financially security when taking the survey (vs. secure) | | | | |  |  |  |  |  | | | |  |  |  | |  |
|  | Financially insecure | 1.4* | **<0.05** | (0.4,2.4) | 1.4** | **<0.01** | (0.6,2.2) | 1.9** | **<0.01** | | | | (0.8,3.0) | 1.6** | **<0.01** | | (0.7,2.6) |
| In lockdown (vs. no) | |  |  |  |  |  |  |  |  | | | |  |  |  | |  |
|  | Yes | 0.9* | **<0.05** | (0.1,1.7) | -0.2 | 0.7 | (-0.8,0.5) | 1.1* | **<0.05** | | | | (0.1,2.0) | 1.0* | **<0.05** | | (0.2,1.8) |
| Days per week having trouble to sleep (vs. zero to two days per week) | | | | | | |  |  |  | | | |  |  |  | |  |
|  | Over two days per week | 2.9*** | **<0.001** | (2.1,3.8) | 3.0*** | **<0.001** | (2.2,3.9) | 5.2*** | **<0.001** | | | | (4.3,6.1) | 3.5*** | **<0.001** | | (2.6,4.5) |
|  | Missing |  |  |  | 0.6 | 0.1 | (-0.2,1.5) |  |  | | | |  | 1.4*** | **<0.01** | | (0.4,2.4) |
| constant | | 4.5*** | **<0.001** | (2.7,6.4) | 3.3* | **<0.05** | (0.4,6.1) | 3.2*** | **<0.001** | | | | (1.8,4.6) | 5.3** | **<0.01** | | (2.1,8.6) |
| Random effects | | **Estimate** | **SE** | **95% CI** | **Estimate** | **SE** | **95% CI** | **Estimate** | | | **SE** | **95% CI** | | **Estimate** | | **SE** | **95% CI** |
| Participant Identity | | 38.5 | 3.0 | (33.0,45.0) | 44.8 | 3.1 | (39.1,51.2) | 45.6 | 3.6 | | | | (39.1,53.2) | 52.4 | 3.8 | | (45.5,60.2) |
| var (residual) | | 22.8 | 1.9 | (19.5,26.8) | 27.5 | 1.2 | (25.2,30.0) | 30.6 | 2.3 | | | | (26.4,35.5) | 38.8 | 1.7 | | (35.6,42.3) |
| Note: This table presents the name of the variables as factor (vs. reference group).* p<.05, ** p<.01, *** p<.001; CI: confidence interval; Tests used: Likelihood Ratio Test and Wald Chi-Squared Test. “Other” work status includes self-employed, carers, and gig workers. | | | | | | | | | | | | | | | | | |
