# Supplementary material for: Coping with COVID-19: a prospective cohort study on young Australians' anxiety and depression symptoms from 2020–2021
Source: Arch Public Health. 2024 Sep 26;82:166. doi: 10.1186/s13690-024-01397-z (PMC11426065; doi:10.1186/s13690-024-01397-z)
Supplement: Supplementary file 3 — Supplementary Material 3. [file 13690_2024_1397_MOESM3_ESM.docx]

| **Additional file 3.**  Mixed-effects of factors associated with DASS-21 scores stratified by state Victoria vs others among young people in Australia 2020-2021 | | | | | | | | | | | | | |
| --- | --- | --- | --- | --- | --- | --- | --- | --- | --- | --- | --- | --- | --- |
| **Potential Risk factor** | | **Anxiety (DASS-21)- Victoria (n=1375)** | | | **Depression (DASS-21)- Victoria (n=1375)** | | | **Anxiety (DASS-21)- Other states (n=1943)** | | | **Depression (DASS-21)- Other states (n=1943)** | | |
|  |  | **β** | **p** | **95% CI** | **β** | **p** | **95% CI** | **β** | **p** | **95% CI** | **β** | **p** | **95% CI** |
| **Age group (vs. 25-29 years)** | |  |  |  |  |  |  |  |  |  |  |  |  |
|  | **15-19** | 3.7 | **0.0** | (1.5,5.9) | 3.0 | **0.0** | (0.5,5.5) | 0.9 | 0.3 | (-0.7,2.6) | 0.5 | 0.6 | (-1.4,2.3) |
|  | **20-24** | 2.0 | **0.0** | (0.6,3.4) | 0.7 | 0.4 | (-0.9,2.2) | 1.2 | **0.0** | (0.2,2.3) | 0.7 | 0.2 | (-0.5,2) |
| **Recruitment type** | |  |  |  |  |  |  |  |  |  |  |  |  |
|  | Pure Profile | -0.5 | 0.5 | (-2,1) | 1.2 | 0.1 | (-0.4,2.8) | 0.2 | 0.8 | (-1.1,1.5) | 0.4 | 0.6 | (-1,1.9) |
| **In lockdown (vs. no)** | |  |  |  |  |  |  |  |  |  |  |  |  |
|  | yes | 0.2 | 0.7 | (-0.6,0.9) | 1.5 | **0.0** | (0.6,2.3) | -0.3 | 0.7 | (-1.4,0.9) | -1.2 | 0.1 | (-2.5,0.2) |
| **Bushfire affected postcode (vs. no)** | |  |  |  |  |  |  |  |  |  |  |  |  |
|  | Yes | 19.9 | **0.0** | (5.9,33.8) | 31.4 | **0.0** | (16.2,46.7) | 0.3 | 0.7 | (-1.1,1.7) | 0.3 | 0.7 | (-1.3,1.9) |
| **Loneliness (vs. less than mild loneliness)** | | | |  |  |  |  |  |  |  |  |  |  |
|  | Moderate loneliness or higher | 4.4 | **0.0** | (3.2,5.6) | 0.3 | 0.9 | (-5,5.5) | 4.1 | **0.0** | (3.2,5) | 3.4 | **0.0** | (0.5,6.4) |
|  | Missing data | 2.0 | 0.5 | (-3.3,7.2) | -0.8 | 0.9 | (-11.4,9.9) | 7.1 | 0.0 | (3.8,10.4) | 0.6 | 0.8 | (-4.2,5.5) |
| **Days per week having trouble to sleep (vs. zero to two days per week)** | | | | |  |  |  |  |  |  |  |  |  |
|  | over 2 days a week | 2.6 | **0.0** | (1.7,3.4) | 0.2 | 0.9 | (-2.1,2.5) | 3.2 | **0.0** | (2.4,4) | 0.6 | 0.5 | (-1.1,2.2) |
|  |  | 0.3 | 0.5 | (-0.6,1.2) | -1.0 | 0.4 | (-3.2,1.2) | 1.4 | **0.0** | (0,2.8) | 0.0 | 1.0 | (-2,2) |
| **Financially security when taking the survey (vs. secure)** | | | | | |  |  |  |  |  |  |  |  |
|  | Financially insecure | 1.3 | **0.0** | (0.4,2.3) | 7.9 | 0.**0** | (6.6,9.3) | 1.6 | **0.0** | (0.7,2.4) | 7.9 | **0.0** | (6.9,9) |
| **Hours spent on social media per day** | | 0.4 | **0.0** | (0.2,0.5) | 3.7 | 0.2 | (-2.1,9.5) | 0.3 | **0.0** | (0.2,0.4) | 7.1 | **0.0** | (3.4,10.8) |
| **Student status (vs. not a current student)** | | | |  |  |  |  |  |  |  |  |  |  |
| Going to school/university/class in person | | 1.9 | **0.0** | (0.4,3.4) | 3.7 | **0.0** | (2.7,4.7) | 2.2 | **0.0** | (1,3.5) | 4.9 | **0.0** | (4,5.8) |
| Studying, by distance/online | | 0.9 | 0.1 | (-0.2,2) | 1.1 | 0.1 | (0,2.2) | 0.2 | 0.8 | (-0.8,1.2) | 4.9 | **0.0** | (3.3,6.6) |
| Deferred, withdrawn, drop out or I don't wish to say | | 3.0 | **0.0** | (1.1,5) | 1.4 | **0.0** | (0.3,2.5) | 1.8 | **0**.0 | (0.1,3.5) | 2.1 | **0.0** | (1.1,3) |
| **LGBTIQA+ (vs. no)** | | | | |  |  |  |  |  |  |  |  |  |
|  | Yes | 2.3 | **0.0** | (1,3.6) | 0.4 | **0.0** | (0.2,0.6) | 1.6 | **0.0** | (0.5,2.6) | 0.3 | **0.0** | (0.2,0.5) |
|  | Missing | 1.8 | 0.5 | (-2.9,6.4) | 2.4 | **0.0** | (0.9,3.8) | -3.7 | 0.1 | (-8,0.6) | 2.2 | **0.0** | (1.1,3.4) |
| **Financially security before the pandemic (vs. secure)** | | | |  |  |  |  |  |  |  |  |  |  |
|  | Financially insecure | 2.0 | **0.0** | (0.2,3.8) | 1.4 | 0.6 | (-3.9,6.7) | 3.3 | **0.0** | (2.1,4.5) | -2.0 | 0.4 | (-6.9,2.9) |
| **Aboriginal or Torres Strait Islander (vs. no)** | | | | |  |  |  |  |  |  |  |  |  |
|  | Yes | 2.9 | 0.2 | (-1.9,7.7) | 0.9 | 0.3 | (-0.6,2.3) | 5.0 | 0.0 | (2.4,7.6) | 0.6 | 0.3 | (-0.6,1.7) |
|  | I don't wish to say and Missing | 1.1 | 0.8 | (-8.5,10.7) | 10.6 | **0.0** | (5.6,15.6) | 3.6 | 0.1 | (-0.7,7.9) | -4.5 | 0.1 | (-9.2,0.2) |
| **Living with (vs. alone)** | | | | |  |  |  |  |  |  |  |  |  |
|  | Parents | -1.8 | 0.1 | (-3.6,0) | 5.5 | 0.5 | (-9.9,21) | -2.8 | **0.0** | (-4.3,-1.3) | -3.8 | 0.1 | (-8.9,1.3) |
|  | Partner | -1.6 | 0.1 | (-3.6,0.3) | 1.7 | 0.1 | (-0.3,3.7) | -3.8 | **0.0** | (-5.4,-2.1) | 2.7 | **0.0** | (1.4,4.1) |
|  | Friends/ roomates | -0.6 | 0.5 | (-2.5,1.3) | -1.5 | 0.1 | (-3.6,0.5) | -2.1 | **0.0** | (-3.8,-0.5) | -1.7 | 0.1 | (-3.4,0.1) |
|  | other | -1.3 | 0.4 | (-4.3,1.7) | -2.4 | **0.0** | (-4.6,-0.2) | -2.5 | **0.0** | (-4.8,-0.1) | -2.4 | 0.0 | (-4.2,-0.5) |
| **Current work status (vs. full-time)** | |  |  |  |  |  |  |  |  |  |  |  |  |
|  | part time | -0.5 | 0.6 | (-2.1,1.1) | -1.0 | 0.4 | (-3.1,1.1) | -1.1 | 0.2 | (-2.7,0.4) | -0.3 | 0.7 | (-2.2,1.5) |
|  | casual | -1.5 | 0.1 | (-3.1,0.2) | -2.2 | 0.2 | (-5.7,1.2) | -1.1 | 0.2 | (-2.7,0.5) | -0.9 | 0.5 | (-3.6,1.7) |
|  | unemployed | -1.4 | 0.1 | (-3.1,0.4) | 0.4 | 0.7 | (-1.9,2.7) | -1.6 | **0.0** | (-3.2,-0.1) | -0.4 | 0.7 | (-2.4,1.7) |
|  | other | -1.0 | 0.4 | (-3.2,1.3) | 0.7 | 0.6 | (-1.6,3) | -0.1 | 0.9 | (-2,1.9) | -0.9 | 0.4 | (-2.9,1) |
| **Work status before the pandemic (vs. full-time)** | | | |  |  |  |  |  |  |  |  |  |  |
|  | part time | -0.3 | 0.8 | (-2.4,1.7) | -1.9 | 0.2 | (-4.6,0.8) | 0.4 | 0.7 | (-1.4,2.2) | -1.7 | 0.1 | (-3.9,0.5) |
|  | casual | -0.7 | 0.5 | (-2.8,1.3) | 2.1 | 0.3 | (-2.1,6.3) | -0.5 | 0.6 | (-2.2,1.3) | -2.2 | 0.1 | (-5.1,0.7) |
|  | unemployed | -0.9 | 0.5 | (-3.4,1.5) | 0.6 | 0.5 | (-1.2,2.3) | -0.1 | 0.9 | (-2,1.8) | 0.9 | 0.2 | (-0.6,2.3) |
|  | other | 2.8 | 0.1 | (-1,6.6) | 0.1 | 0.9 | (-1.1,1.4) | -0.6 | 0.6 | (-3.2,1.9) | -0.2 | 0.8 | (-1.3,1) |
| **Gender (vs. male)** | | |  |  |  |  |  |  |  |  |  |  |  |
|  | female | 1.2 | 0.1 | (-0.1,2.5) | 2.0 | 0.1 | (-0.2,4.2) | 0.6 | 0.3 | (-0.5,1.6) | 2.8 | **0.0** | (0.8,4.7) |
|  | nonbinary | 9.8 | **0.0** | (5.3,14.4) | -0.1 | 0.9 | (-2.2,1.9) | 1.3 | 0.5 | (-2.9,5.6) | -1.2 | 0.1 | (-2.5,0.2) |
|  | other | -0.9 | 0.9 | (-15,13.2) | 0.5 | 0.9 | (-6.6,7.6) | -1.0 | 0.7 | (-5.6,3.6) | 0.9 | 0.7 | (-3.8,5.6) |
| **In a relationship (vs. no)** | | | |  |  |  |  |  |  |  |  |  |  |
|  | Yes | 0.5 | 0.4 | (-0.7,1.6) | 0.7 | 0.5 | (-1.2,2.5) | 1.7 | **0.0** | (0.7,2.6) | -0.6 | 0.5 | (-2.4,1.2) |
|  | Prefer not to say/ missing | -5.3 | 0.1 | (-12,1.3) | 0.9 | 0.3 | (-1,2.8) | 2.5 | 0.3 | (-1.8,6.8) | 0.7 | 0.4 | (-1.1,2.5) |
| **Highest completed or enrolled level of education at baseline (vs. high school)** | | | | | |  |  |  |  |  |  |  |  |
|  | Tertiary | 0.3 | 0.8 | (-1.6,2.1) | 2.1 | **0.0** | (0.1,4.1) | -0.6 | 0.3 | (-1.8,0.6) | 1.7 | 0.1 | (0,3.5) |
|  | Missing | -0.9 | 0.8 | (-7.3,5.5) | 2.4 | 0.1 | (-0.1,5) | 0.1 | 1.0 | (-4.1,4.3) | 2.3 | **0.0** | (0.1,4.6) |
| **Residential status in Australia (vs. citizen)** | | | | |  |  |  |  |  |  |  |  |  |
|  | Permanent Resident | 2.1 | **0.0** | (0,4.1) | 0.3 | 0.6 | (-1,1.6) | 0.3 | 0.6 | (-1.1,1.8) | 1.1 | 0.1 | (0,2.1) |
|  | Other Temporary visa | 0.0 | 1.0 | (-2,2) | -5.0 | 0.2 | (-12.6,2.6) | -0.3 | 0.7 | (-2.1,1.5) | 0.6 | 0.8 | (-4.3,5.6) |
|  | _cons | 3.2 | 0.0 | (0.4,5.9) | 4.0 | **0.0** | (1,7.1) | 5.1 | 0.0 | (3.2,7) | 5.7 | **0.0** | (3.6,7.8) |
| **Random-effects** | | **Estimate** | **SE** | **95% CI** | **Estimate** | **SE** | **95% CI** | **Estimate** | **SE** | **95% CI** | **Estimate** | **SE** | **95% CI** |
| Participant Identity | | 41.8 | 3.3 | (35.8,48.8) | 48.5 | 3.9 | (41.4,56.8) | 38.0 | 2.6 | (33.1,43.5) | 43.7 | 3.3 | (37.7,50.7) |
| var(Residual) | | 24.7 | 1.4 | (22.1,27.5) | 34.0 | 1.8 | (30.6,37.8) | 28.1 | 1.5 | (25.3,31.2) | 39.8 | 2.1 | (35.9,44.1) |
| **Note**: This table presents the name of the variables as factor (vs. reference group).* p<.05, ** p<.01, *** p<.001; CI: confidence interval; Tests used: Likelihood Ratio Test and Wald Chi-Squared Test. “Other” work status includes self-employed, carers, and gig workers. | | | | | | | | | | | | | |
