# Supplementary material for: Coping with COVID-19: a prospective cohort study on young Australians' anxiety and depression symptoms from 2020–2021
Source: Arch Public Health. 2024 Sep 26;82:166. doi: 10.1186/s13690-024-01397-z (PMC11426065; doi:10.1186/s13690-024-01397-z)
Supplement: Supplementary file 4 — Supplementary Material 4. [file 13690_2024_1397_MOESM4_ESM.docx]

**Additional file 4**

| **Additional file 4.a** Sensitivity Analysis: Logistic Regression Predicting Completion of All Four Surveys in Young Australians Using DASS-21 Anxiety Baseline Scores (n=1936) | | | |
| --- | --- | --- | --- |
|  | **Completion of all 4 surveys (Y/N)**  **N=1936** | | |
|  | **β-coefficient** | **p-value** | **(95% CI)** |
|  |  |  |  |
| **DASS-21 Anxiety Baseline scores** | 0.0 | 0.1 | (0,0) |
|  |  |  |  |
| **Age group at baseline (vs. 25–29 years)** |  |  |  |
| 15-19 | -1.1 | 0.0 | (-1.8,-0.4) |
| 20-24 | -0.7 | 0.0 | (-1.1,-0.2) |
|  |  |  |  |
| **Recruitment approach (vs. research market panel)** |  |  |  |
| Social Media | 2.3 | 0.0 | (1.6,3.1) |
|  |  |  |  |
| **In lockdown (vs. no)** |  |  |  |
| yes | 0.0 | 1.0 | (-0.3,0.3) |
|  |  |  |  |
| **Bushfire affected postcode (vs. no)** |  |  |  |
| Yes | -0.3 | 0.4 | (-1,0.4) |
|  |  |  |  |
| **Loneliness (vs. mild loneliness or lower)** |  |  |  |
| Moderate loneliness | 0.1 | 0.4 | (-0.2,0.5) |
| Missing data | -0.4 | 0.6 | (-1.9,1.2) |
|  |  |  |  |
| **Days per week having trouble to sleep (vs. zero to two days per week)** |  |  |  |
| over 2 days a week | -0.1 | 0.7 | (-0.8,0.5) |
| Missing | -0.4 | 0.4 | (-1.2,0.5) |
|  |  |  |  |
| **Financial security when taking the survey (vs. secure)** |  |  |  |
| Financially insecure | -0.2 | 0.3 | (-0.6,0.2) |
|  |  |  |  |
| **Hours spent on social media per day** | 0.0 | 0.2 | (-0.1,0) |
|  |  |  |  |
| **Student status (vs. not a current student)** |  |  |  |
| Going to school/un. | -0.1 | 0.8 | (-1.1,0.8) |
| Studying, by distance | 0.4 | 0.1 | (-0.1,0.8) |
| Deferred, withdraw. | -1.1 | 0.1 | (-2.1,0) |
|  |  |  |  |
| **LGBTQIA+ (vs. no)** |  |  |  |
| LGBTIQA+ | 0.2 | 0.2 | (-0.1,0.6) |
| Missing | -0.6 | 0.6 | (-2.8,1.5) |
|  |  |  |  |
| **Financial security before the pandemic (vs. secure)** |  |  |  |
| Financially insecure | 0.3 | 0.2 | (-0.2,0.8) |
|  |  |  |  |
| **Aboriginal or Torres Strait Islander (vs. no)** |  |  |  |
| Yes | -0.2 | 0.8 | (-1.5,1.1) |
| I don't wish to say | 0.1 | 1.0 | (-2.1,2.3) |
|  |  |  |  |
| **Living with (vs. alone)** |  |  |  |
| Parents | 0.1 | 0.7 | (-0.5,0.8) |
| Partner | 0.1 | 0.7 | (-0.6,0.8) |
| Friends/ roomates | -0.1 | 0.7 | (-0.8,0.6) |
| other | 0.1 | 0.9 | (-1,1.2) |
|  |  |  |  |
| **Current work status (vs. full-time)** |  |  |  |
| part time | -0.1 | 0.7 | (-0.9,0.6) |
| casual | 0.2 | 0.7 | (-0.6,1) |
| unemployed | 0.1 | 0.8 | (-0.7,0.9) |
| other | 0.1 | 0.9 | (-0.8,1) |
|  |  |  |  |
| **Work status before the pandemic (vs. full-time)** |  |  |  |
| part time | 0.0 | 0.9 | (-0.7,0.8) |
| casual | 0.2 | 0.5 | (-0.5,1) |
| unemployed | 0.0 | 0.9 | (-0.9,0.8) |
| other | 0.5 | 0.4 | (-0.6,1.5) |
|  |  |  |  |
| **Gender (vs. male)** |  |  |  |
| female | 0.6 | 0.0 | (0.2,1) |
| nonbinary | 0.0 | 1.0 | (-1.3,1.3) |
| other | 0.9 | 0.3 | (-0.8,2.6) |
|  |  |  |  |
| **In a relationship (vs. no)** |  |  |  |
| Yes | -0.4 | 0.1 | (-0.7,0) |
| Prefer not to say/. | -0.5 | 0.7 | (-2.7,1.8) |
|  |  |  |  |
| **Highest completed or enrolled level of education at baseline (vs. high school)** |  |  |  |
| Tertiary | -0.2 | 0.6 | (-0.7,0.4) |
| Missing | -0.4 | 0.8 | (-2.6,1.9) |
|  |  |  |  |
| **Residential status in Australia (vs. citizen)** |  |  |  |
| Permanent Resident | -0.3 | 0.3 | (-0.9,0.3) |
| Other Temporary visa | 0.1 | 0.9 | (-0.6,0.7) |
|  |  |  |  |
| **Constant** | -3.0 | 0.0 | (-3.9,-2.2) |

| **Additional file 4.a** Sensitivity Analysis: Logistic Regression Predicting Completion of All Four Surveys in Young Australians Using DASS-21 Depression Baseline Scores (n=1936) | | | |
| --- | --- | --- | --- |
|  | **Completion of all 4 surveys (Y/N)**  **N=1936** | | |
|  | **β-coefficient** | **p-value** | **(95% CI)** |
|  |  |  |  |
| **DASS-21 Depression Baseline Score** | 0.0 | 0.9 | (0.0,0.0) |
|  |  |  |  |
| **Age group at baseline (vs. 25–29 years)** |  |  |  |
| 15-19 | -1.2 | 0.0 | (-1.9,-0.4) |
| 20-24 | -0.7 | 0.0 | (-1.1,-0.2) |
|  |  |  |  |
| **Recruitment approach (vs. research market panel)** |  |  |  |
| Social Media | 2.3 | 0.0 | (1.5,3.1) |
|  |  |  |  |
| **In lockdown (vs. no)** |  |  |  |
| yes | 0.0 | 1.0 | (-0.3,0.3) |
|  |  |  |  |
| **Bushfire affected postcode (vs. no)** |  |  |  |
| Yes | -0.3 | 0.4 | (-1,0.4) |
|  |  |  |  |
| **Aboriginal or Torres Strait Islander (vs. no)** |  |  |  |
| Yes | -0.3 | 0.7 | (-1.6,1) |
| I don't wish to say and Missing | 0.0 | 1.0 | (-2.2,2.2) |
|  |  |  |  |
| **Residential status in Australia (vs. citizen)** |  |  |  |
| Permanent Resident | -0.3 | 0.3 | (-0.9,0.3) |
| Other Temporary visa | 0.1 | 0.9 | (-0.6,0.7) |
|  |  |  |  |
| **Loneliness (vs. mild loneliness or lower)** |  |  |  |
| Moderate loneliness or higher | 0.1 | 0.7 | (-0.3,0.4) |
| Missing data | -0.4 | 0.6 | (-2,1.1) |
|  |  |  |  |
| **Days per week having trouble to sleep (vs. zero to two days per week)** |  |  |  |
| over 2 days a week | -0.2 | 0.6 | (-0.8,0.5) |
| Missing | -0.4 | 0.4 | (-1.2,0.5) |
|  |  |  |  |
| **Financial security when taking the survey (vs. secure)** |  |  |  |
| Financially insecure | -0.3 | 0.2 | (-0.6,0.1) |
|  |  |  |  |
| **Hours spent on social media per day** | 0.0 | 0.2 | (-0.1,0) |
|  |  |  |  |
| **LGBTQIA+ (vs. no)** |  |  |  |
| LGBTIQA+ | 0.2 | 0.2 | (-0.1,0.5) |
| Missing | -0.5 | 0.6 | (-2.7,1.6) |
|  |  |  |  |
| **Gender (vs. male)** |  |  |  |
| female | 0.6 | 0.0 | (0.2,1) |
| nonbinary | -0.1 | 0.9 | (-1.4,1.2) |
| other | 0.9 | 0.3 | (-0.8,2.6) |
|  |  |  |  |
| **Financial security before the pandemic (vs. secure)** |  |  |  |
| Financially insecure | 0.3 | 0.3 | (-0.2,0.8) |
|  |  |  |  |
| **Living with (vs. alone)** |  |  |  |
| Parents | 0.2 | 0.6 | (-0.5,0.8) |
| Partner | 0.2 | 0.6 | (-0.5,0.9) |
| Friends/ roomates | -0.1 | 0.7 | (-0.8,0.6) |
| other | 0.1 | 0.8 | (-1,1.2) |
|  |  |  |  |
| **Work status before the pandemic (vs. full-time)** |  |  |  |
| part time | 0.0 | 0.9 | (-0.7,0.8) |
| casual | 0.3 | 0.5 | (-0.5,1) |
| unemployed | 0.0 | 1.0 | (-0.9,0.8) |
| other | 0.5 | 0.4 | (-0.6,1.6) |
|  |  |  |  |
| **Student status (vs. not a current student)** |  |  |  |
| Going to school/university/class in person | -0.2 | 0.7 | (-1.2,0.8) |
| Studying, by distance/online | 0.3 | 0.1 | (-0.1,0.7) |
| Deferred, withdrawn, drop out or I don't wish to say | -1.1 | 0.1 | (-2.2,0) |
|  |  |  |  |
| **Highest completed or enrolled level of education at baseline (vs. high school)** |  |  |  |
| Tertiary | -0.1 | 0.6 | (-0.7,0.4) |
| Missing | -0.3 | 0.8 | (-2.6,1.9) |
|  |  |  |  |
| **Current work status (vs. full-time)** |  |  |  |
| part time | -0.1 | 0.7 | (-0.8,0.6) |
| casual | 0.2 | 0.7 | (-0.6,1) |
| unemployed | 0.1 | 0.8 | (-0.7,0.9) |
| other | 0.0 | 0.9 | (-0.9,0.9) |
|  |  |  |  |
| **In a relationship (vs. no)** |  |  |  |
| Yes | -0.4 | 0.1 | (-0.8,0) |
| Prefer not to say/ missing | -0.5 | 0.7 | (-2.8,1.8) |
|  |  |  |  |
| **Constant** | -3.1 | 0.0 | (-3.9,-2.2) |
