# Supplementary material for: Coping with COVID-19: a prospective cohort study on young Australians' anxiety and depression symptoms from 2020–2021
Source: Arch Public Health. 2024 Sep 26;82:166. doi: 10.1186/s13690-024-01397-z (PMC11426065; doi:10.1186/s13690-024-01397-z)
Supplement: Supplementary file 5 — Supplementary Material 5. [file 13690_2024_1397_MOESM5_ESM.docx]

**Additional file 5.** Frequency of Employment Status from April 2020 to August 2021 of participants who experienced an employment change (n=931)

| **Frequency of Employment Status Changes from April 2020 to August 2021 n(%)** | | | | | | |
| --- | --- | --- | --- | --- | --- | --- |
| **Timepoint** | **Full time** | **Part time** | **casual** | **unemployed** | **other** | **Total** |
| 1 | 23(7.5) | 46(15) | 48(15.7) | 154(50.3) | 35(11.4) | 306(100) |
|  |  |  |  |  |  |  |
|  |  |  |  |  |  |  |
| 2 | 33(15.3) | 32(14.8) | 66(30.6) | 60(27.8) | 25(11.6) | 216(100) |
|  |  |  |  |  |  |  |
|  |  |  |  |  |  |  |
| 3 | 39(18) | 28(12.9) | 87(40.1) | 44(20.3) | 19(8.8) | 217(100) |
|  |  |  |  |  |  |  |
|  |  |  |  |  |  |  |
| 4 | 36(18.8) | 38(19.8) | 85(44.3) | 18(9.4) | 15(7.8) | 192(100) |
| Total | 131(14.1) | 144(15.5) | 286(30.7) | 276(29.7) | 94(10.1) | **931**(100) |
